# A Holistic Approach to Evaluating Cellular Communication Pathways

**DOI:** 10.1371/journal.pbio.0020085

**Published:** 2004-03-16

To function properly, cells must keep constant tabs on the environmental conditions around them, such as the presence of growth hormones in the blood or the proximity of neighboring cells. These external cues are relayed into the cell through a cascade of chemical and physical reactions referred to as signal transduction. Signal transduction pathways inform and regulate almost all activity within the cell, from protein production to cell division. Understanding these processes is fundamental to biology, but the sheer number of molecules and interactions in some pathways makes thorough documentation difficult.

Taking a holistic approach that combines both computational models and experimental manipulations, scientists have described the web of interactions involved in the aryl hydrocarbon receptor (AHR) signal transduction pathway. AHR belongs to the Per–Arnt–Sim (PAS) superfamily of sensor molecules that regulate functions like development, the sleep-wake cycle, and cellular reaction to oxygen deprivation. Unlike many receptors that are embedded in the cell membrane, AHR floats freely in the main body of the cell, called the cytosol. There it waits for a stimulus or ligand, such as a dioxin molecule, to enter the cell and bind to it. Once bound, AHR undergoes a host of changes, glomming on to additional molecules before it enters the cell nucleus and acts as a transcription factor, initiating the production of enzymes to digest foreign, or xenobiotic, compounds. The AHR pathway is a curiosity; though found in all vertebrates, the natural, or endogenous, ligand remains unknown. Without this knowledge, researchers are limited in the kind of experiments they can perform to evaluate the pathway.[Fig pbio-0020085-g001]

**Figure pbio-0020085-g001:**
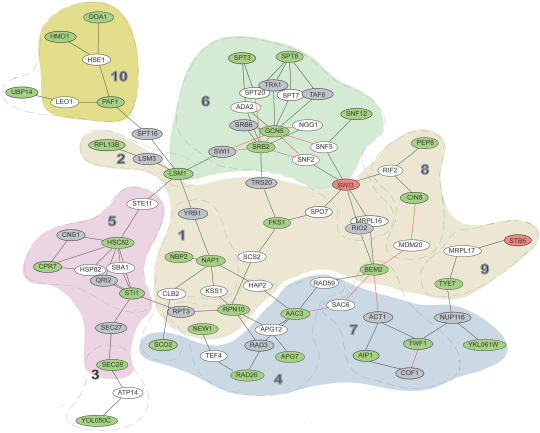
Protein-interactive-network for AHR signaling

Christopher Bradfield and colleagues used yeast as a model system to elucidate the steps involved in this pathway, which regulates vertebrate cell response to pollutants like dioxins. To first assess the molecules involved in the AHR pathway, the team used 4,507 yeast “deletion” strains, each strain missing one gene from its genome. They then inserted the AHR gene into the strains using small rings of movable DNA called plasmids. Though yeast does not naturally possess AHR, it is an ideal genetic model for studying signaling pathways due to its quick generation time, small, well-characterized genome, and similarity to vertebrate systems.

Bradfield's team exposed each strain to a receptor stimulus or agonist and screened them for AHR response. If a deletion strain showed significantly reduced activity, they concluded that the missing gene was a key component to the signal pathway. The researchers identified 54 genes that had a significant influence on AHR response. Only two of these genes, termed modifiers, had been previously identified.

Signaling pathways usually boil down to a series of discrete steps. To identify steps of the AHR pathway, the researchers constructed a spider web-like map called a "protein interaction network," or PIN, based on previously known interactions between the proteins encoded by the 54 modifier genes. The resulting map revealed groups of highly connected, related modifiers, which the authors proposed to be steps in the pathway. Though other studies have used the newly developed PIN strategy to investigate cellular processes, Bradfield's team also annotated their PIN through a series of experiments both to support the identity of and to better understand the protein groups, referred to as functional modules.

With tests based on discrete receptor signaling events, known active structural regions, reaction to different types and concentrations of agonists, and functional location within the cell, Bradfield's team organized the functional modules into five steps. One group of modifiers is involved in AHR folding, the conformational change that occurs when the receptor binds to a toxin. With the help of other modifiers, the new AHR complex is then translocated into the cell nucleus. Once in the nucleus, a series of modifiers assist the AHR in its role as a transcription factor. The researchers also identified a step in the pathway that controls production of AHR itself and another unknown "step" that takes place inside the nucleus.

As AHR is thought to be a prototype PAS receptor, understanding the steps in this pathway will likely guide future research on the entire family, allowing scientists to study in detail individual steps in these complex pathways. The highly integrated method reported here could also be used to study most other mammalian signaling pathways, giving scientists a new tool as they attempt to understand how cells respond to their changing environment.

